# 

**Published:** 1895-05-18

**Authors:** 


### CONTENTS.

Some Points in the Midwives Bill - - - - - - - 107

Recent Science - - - - - - - - - - - 108

Pulmonary Congestion and its Treatment. II.—By Sir B. W. Richardson, M.D., F.R.S. - - - - - - - - 109

Literature for the Million - - - - - - - - 110

Notes and News - - - - - - - - - - - 112

Medical Progress and Hospital Clinics—

Cataract as it is met with in Ordinary Practice - - - - 113

Syphilitic Disease in the Spinal Column - - - - - 114

Progress in Medicine.—Rheumatic Affections - - - 115

Progress in Surgery.—Surgery of the Œsophagus and Stomach 116

Annotations.—The Moral of the Cab-call—A Toothache Benefactor—A Bristol Philosopher, or What? - - - - - 111

Around the Hospitals - - - - - - - - - - 118

The Institutional Workshop.—

Hospital Construction.—The Adelaide Hospital, Melbourne, South Australia - - - - - - - - - - 119

Hospital Festivals and Meetings - - - - - - - 120

Notes and Queries - - - - - - - - - - 120

The Book World of Medicine and Science - - - - 121

Scraps and Gleanings - - - - - - - - - 121

The Student of Medicine - - - - - - - - - 122

### EXTRA SUPPLEMENT.—THE NURSING MIRROR.

News from the Nursing World.—Invalid Children—Cauls and Coins—A Worthy Society—Appreciative East-enders—A Crusade Against Caps—National Health Society—Practical Missionaries—District Nursing at Haverfordwest—Royal British Nurses Association—St. Patrick's Home—Indirect Payment for Honorary (?) Services — Women Students in India—Short Items - - - - - - - xlv

Elementary Anatomy and Surgery for Nurses. By W. McAdam Eccles, M.B., M.S., F.R.C.S. - - - - - - xlvii

Dundee Royal Infirmary - - - - - - - - - xlvii

Lewes District Nurse Fund - - - - - - - - xlvii

The Metropolitan Nursing Association - - - - xlviii

Our American Letter—Minor Appointments - - - xlviii

Letters from Upper Burma. By Mrs. Ernest Hart. II.—Lepers and Leper Homes - - - - - - - - - l

Care of the Sick in Alexandria and Cairo. I.—The Native Hospital - - - - - - - - - - - - l

Everybody's Opinion—Where to Go - - - - - li

With St. Peter's Society to Meiringen - - - - - lii

Appointments—Notes and Queries - - - - - - lii

